# Agreement Among Paper and Electronic Modes of the EQ-5D-5L

**DOI:** 10.1007/s40271-020-00419-6

**Published:** 2020-04-28

**Authors:** J. Jason Lundy, Stephen Joel Coons, Emuella Flood, Mira J. Patel, the ePRO Consortium

**Affiliations:** 1 Outcometrix, 433 Central Avenue, Suite 300, St. Petersburg, FL 33701 USA; 2 Patient-Reported Outcome Consortium, Critical Path Institute, Tucson, AZ USA; 3 AstraZeneca, Gaithersburg, MD USA; 4 Division of Clinical Outcome Assessment, Office of New Drugs, Center for Drug Evaluation and Research, Food and Drug Administration, White Oak, MD USA

## Abstract

**Introduction:**

While the EQ-5D-5L has been migrated to several electronic modes, evidence supporting the measurement equivalence of the original paper-based instrument to the electronic modes is limited.

**Objectives:**

This study was designed to comprehensively examine the equivalence of the paper and electronic modes (i.e., handheld, tablet, interactive voice response [IVR], and web).

**Methods:**

As part of the foundational work for this study, the test–retest reliability of the paper-based, UK English format of the EQ-5D-5L was assessed using a single-group, single-visit, two-period, repeated-measures design. To compare paper and electronic modes, three independent samples were recruited into a three-period crossover study. Each participant was assigned to one of six groups to account for order effects. Descriptive statistics, mean differences (i.e., split-plot analysis of variance [ANOVA]), and intraclass correlation coefficients (ICCs) were calculated.

**Results:**

The test–retest results showed mean differences near zero and ICC values > 0.90 for both the index and the EQ VAS scores. For the electronic comparisons, mean difference confidence intervals (CIs) for the EQ-5D index scores and EQ VAS scores reflected equivalence of the means across all modes, as the CIs were wholly contained inside the equivalence interval. Further, the ICC 95% lower CIs for the index and EQ VAS scores showed values above the thresholds for denoting equivalence across all comparisons in each sample. No significant mode-by-order interactions were present in any ANOVA model.

**Conclusions:**

Overall, our comparisons of the paper, screen-based, and phone-based formats of the EQ-5D-5L provided substantial evidence to support the measurement equivalence of these modes of data collection.

## Key Points for Decision Makers

**Table:** 

The EQ-5D-5L has been migrated to several electronic modes of data collection. This study aimed to determine the measurement equivalence of the original paper-based instrument versus all available electronic modes.
This study comprehensively examined the equivalence of the EQ-5D-5L on all available electronic modes (i.e., handheld, tablet, interactive voice response, and web).
The comparisons of the paper format and the screen-based and phone-based formats of the EQ-5D-5L provided substantial evidence supporting the measurement equivalence of these modes of data collection.

## Introduction

Assessing the measurement equivalence of paper-based instruments compared with the electronic data collection modes to which they have been migrated is recommended to ensure the comparability of scores between the electronic and paper-based modes [1]. Furthermore, assessing equivalence between and among various electronic modes provides additional evidence supporting the use of a particular instrument on multiple data collection modes, which can be beneficial to researchers who wish to use the measure among the various electronic modes [2]. The EQ-5D-5L is a patient-reported outcome (PRO) instrument commonly used in clinical trials to assess health-related quality of life [3]. The EQ-5D-5L has been migrated to several electronic modes of data collection [4], but there is a dearth of evidence supporting the measurement equivalence of the original paper-based instrument compared with those electronic modes. This study was designed to document the test–retest reliability of the paper-based EQ-5D-5L and comprehensively examine the equivalence of the available electronic modes (i.e., handheld, tablet, interactive voice response [IVR], and web). Hence, the overall aim of this study was to generate empirical evidence regarding the measurement equivalence of data collected via various modes for the EQ-5D-5L.

## Methods

### Study Assessment

The EQ-5D-5L is a 6-item generic measure of health status. Responses for the five-item descriptive system are assessed using a five-level verbal rating scale (VRS), and a single overall health status item is measured on a numeric rating scale from 0 to 100 (EQ VAS). Each of the items reference the participant’s health at the time of assessment (i.e., “today”). Two scores are produced: a population preference-based index value (i.e., EQ-5D index) based on the five descriptive items and a health status score based on the global EQ VAS item [5]. The EQ-5D-5L was the only assessment included in this study and was completed in person by participants at market research interview facilities in the UK.

### Study Design

As part of the foundational work for this study, the test–retest reliability of the paper-based UK English format of the EQ-5D-5L was assessed using a single-group, single-visit, two-period, repeated-measures design. In total, 60 participants were recruited to complete one paper assessment of the EQ-5D-5L, followed by a 30-min distraction task (e.g., Sudoku or crossword puzzles, reading, or watching TV), and then the second paper assessment of the EQ-5D-5L. The distraction task was included in an attempt to wash out any memory effect that may bias the results of the study. Descriptive statistics, mean differences, and intraclass correlation coefficient (ICC) were calculated. These data formed the basis upon which we compared the data generated by the electronic modes of the EQ-5D-5L.

**Fig. 1:**
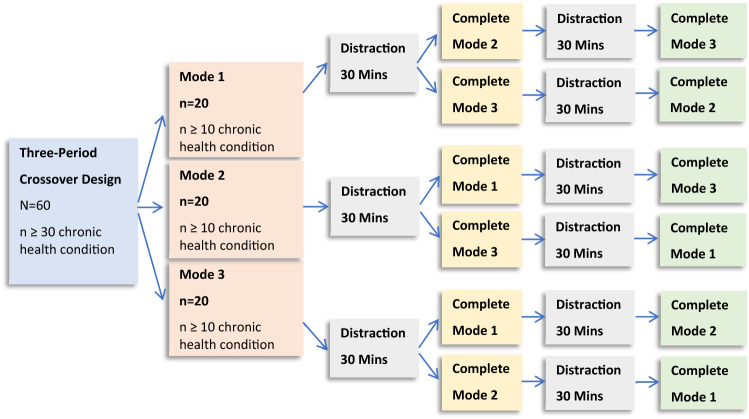
Equivalence study scheme used in the four crossover comparisons independent sample

To compare the paper and electronic modes, four independent samples were recruited into a three-period crossover study (Fig. 1). Each participant, from their respective study sample, was assigned to one of six groups for which the order of administration of the modes being compared was varied to account for order effects. Between each administration of the EQ-5D-5L, participants were instructed to perform a distraction task for 30 min (as described in the previous paragraph). Each scheme compared electronic and paper modes to test for measurement equivalence (see Sect. 2.5). These data and the test–retest data were analyzed in a similar manner (mean differences and ICC).

Finally, to address whether the data were equivalent between various electronic modes, one independent sample of 60 participants was recruited into a three-period crossover study. The participants completed three different electronic modes (handheld, tablet, web) with varying orders of administration, similar to the other designs. A distraction task (as previously described) was included between each administration to wash out any memory effect.

All participants received a written informed consent form that they reviewed with study staff and signed before participating in the study. This study was reviewed by and conducted under the auspices of Salus IRB (Austin, TX, USA).

### Sample Size

The sample size target (*n* = 60) for the independent samples was based on the sample size calculations for comparisons involving the ICC from Streiner and Norman [6] and Bonett [7].

From Streiner and Norman [6]:

$$ n \, = \, 2 \, + \, \left[ {k/2\left( {k - 1} \right)\left( {ZR - ZR^{ - } } \right)^{2} } \right], $$

where *k* is the number of assessments; *ZR* = 1/2log[1 + (*k* − 1)*R*/1 − *R*]; *R* is the specified value of the reliability coefficient; *ZR*^−^ = 1/2log[1 + (*k* − 1)*R*^−^/1 − *R*^−^]; *R*^−^ = *R* − SE; and SE is the standard error, or half of the one-sided confidence interval (CI).

This calculation produced a sample size of 46 based on a target reliability of 0.80, a minimum reliability threshold of 0.70, and a 95% CI across three administrations.

$$ N = 2 + [3/\left( 2 \right)\left( 2 \right)\left( {1.2825 - 1.1513} \right)^{2}] = 46. $$

From Bonett [7]:

$$ n \, = \, \left\{ \left[8z^{2}_{\alpha /2} (1 - \rho )^{2} ( {1 + ( {k - 1} )\rho^{2}) } \right]/\left[ {k( {k - 1} )\omega^{2} } \right]\right\} + 1, $$

where *k* is the number of assessments, *ρ* is the specified value of the reliability coefficient, and *ω* is the width of the 95% CI.

This calculation produced a sample size of 36 based on a target reliability of 0.80, two-sided CI width (*ω*) of 0.20, and a 95% CI across three administrations.

$$ n = \left\{ \left[8\left(1.96^{2} \right) \left(0.2^{2}\right)\left( 1 + (2)\left(0.8^{2}\right)\right) \right]\left[3(2)\left(0.2^{2}\right)\right] \right\}  + 1 = 36. $$

### Recruitment Criteria

All participants were recruited as a convenience sample from the UK general population, were aged ≥ 18 years, and fluent in English. In an attempt to increase the variability in scores, at least 50% of each sample had a chronic health condition that caused daily pain or discomfort, depression or anxiety, or problems dressing, washing, walking, or performing usual activities; there were no quotas for specific types of chronic health conditions. Sample diversity was sought with respect to age, sex, and education level. All participants were recruited using internet and newspaper advertising. Participants were informed that the purpose of the study was to test a general health questionnaire in different formats (paper and electronic) but were not instructed to provide similar responses or told that their responses would be examined for agreement.

### Quantitative Analysis: General Guidelines

The following guidelines were applied to the analyses of the EQ-5D-5L scores:

- Continuous variables are described by their mean and standard deviation (SD). The EQ-5D-5L index scores and EQ VAS responses were treated as interval-level data.
- Categorical variables are described by their frequency and percentages. Categorical variables include the five health state items with VRS response categories and were treated as ordinal-level data.
- ICCs were used to measure the strength of relationship between continuous variables, and kappa correlation coefficients were used for the categorical variables, where appropriate (i.e., test–retest data). Quadratic weights were used for the computation of weighted kappa [8–10].
- A variable for the order of administration, ranging from one to six to represent the six possible mode combination orders, was derived for use in the three-period repeated-measures analyses to test the mode-by-order interaction.
- EQ-5D-5L index values were based on the crosswalk calculations from the three-level EQ-5D (EQ-5D-3L) in the UK population [11].
- No imputation was made for missing data.
- Equivalence analyses relied on evaluating the strength of agreement between variables using the ICC and the CIs of mean differences. Because the roles of alpha and beta are reversed in the equivalence paradigm, significance testing was only used to evaluate the order-by-mode interaction (*p* ≤ 0.05), where applicable [12].
- No adjustments for multiplicity were made.
- All analyses were performed using R version 3.3.1 and SPSS version 24.

Testing of the mean differences was based on analysis of variance (ANOVA) (i.e., split-plot ANOVA) with factors for participant, mode of data collection, and order [13]. The means, SDs, mean differences and 95% CI, and *p *values from the significance tests of the order-by-mode interaction for the EQ-5D-5L index scores and EQ VAS scores are reported. The mean differences in the three-period comparisons were evaluated using an equivalence threshold based on half SD of the paper-based EQ-5D-5L index and EQ VAS scores from the test–retest component of the study. To analyze the reproducibility of the measurement between the various data collection modes, correlational analyses based on the ICC of EQ-5D-5L index scores and EQ VAS scores were conducted. The ICC (ICC 3,1 based on Shrout and Fleiss [8]) was calculated based on the same ANOVA model previously described; this analysis is the same regardless of whether the data collection mode is treated as a fixed or random effect. The ICCs were evaluated for equivalence by comparing the lower bound of the ICC 95% CI to the thresholds established in the test–retest component of the study (i.e., the paper-based ICC 95% CI lower bound) [14]. Kappa correlation coefficients were used for the categorical variables, where appropriate (i.e., test–retest data) [9]. Quadratic weights were used for the computation of weighted kappa [10].

## Results

### Paper Test–Retest Results

#### Sample Description

We included 60 participants: 39 females and 21 males (Table 1). The average ± SD age among all participants was 46.3 ± 15.1 years (range 21–86). A range of educational attainment was present in the sample, with most (*n* = 34) participants completing either an undergraduate or postgraduate degree, and nine participants attending college/technical college. Most participants (*n* = 38) were English/Welsh/Scottish/Northern Irish/British, nine were Black/African/Caribbean/Black British, four were Indian, and the remaining nine participants were distributed among various ethnic groups at rates ≤ 5%.

**Table 1:** Demographic characteristics

Characteristics	Paper test–retest (*n* = 60)	Paper–handheld–web (*n* = 60)	Paper–handheld–tablet (*n* = 60)	Paper–web–IVR (*n* = 61)	Handheld–tablet–web (*n* = 60)
Sex					
Male	21 (35)	27 (45)	31 (51.67)	13 (21.3)	25 (58.33)
Female	39 (65)	33 (55)	29 (48.33)	48 (78.7)	35 (41.67)
Age	46.3 ± 15.1 (21–86)	46.5 ± 12.6 (20–79)	43.9 ± 14.0 (22–82)	49.9 ± 15.8 (18–77)	38.2 ± 13.6 (18–72)
Education					
Left school with no qualifications	1 (1.67)	1 (1.67)	1 (1.67)	2 (3.3)	1 (1.67)
GCSE or equivalent	9 (15.0)	7 (11.67)	2 (3.33)	8 (13.1)	7 (11.67)
A level or equivalent	7 (11.67)	9 (15.0)	6 (10.0)	8 (13.1)	10 (16.67)
College/technical college	9 (15.0)	14 (23.33)	7 (11.67)	11 (18.0)	9 (15.0)
University: undergraduate level	25 (41.67)	21 (35.0)	25 (41.67)	20 (32.8)	19 (31.67)
University: postgraduate level	9 (15.0)	8 (13.33)	19 (31.67)	12 (19.7)	14 (23.33)
Ethnic group					
African	–	1 (1.67)	–	2 (3.3)	2 (3.33)
Asian/Asian British	1 (1.67)	2 (3.33)	4 (6.67)	5 (8.2)	1 (1.67)
Bangladeshi	2 (3.33)	1 (1.67)	3 (5.0)	–	1 (1.67)
Black/African/Caribbean/Black British	9 (15.0)	4 (6.67)	5 (8.33)	–	6 (10.0)
Caribbean	–	–	1 (1.67)	–	2 (3.33)
Chinese	–	1 (1.67)	–	–	–
English/Welsh/Scottish/Northern Irish/British	38 (63.33)	32 (53.33)	22 (36.67)	53 (86.9)	26 (43.33)
Indian	4 (6.67)	3 (5.0)	2 (3.33)	–	1 (1.67)
Irish	–	1 (1.67)	–	1 (1.6)	1 (1.67)
Mixed/multiple ethnic groups	2 (3.33)	3 (5.0)	6 (10.0)	–	5 (8.33)
Other	3 (5.0)	7 (11.67)	17 (28.33)	–	9 (15.0)
Pakistani	–	1 (1.67)	–	–	2 (3.33)
White and Asian	1 (1.67)	–	–	–	1 (1.67)
White and Black African	–	2 (3.33)	–	–	3 (5.0)
White and Black Caribbean	–	2 (3.33)	–	–	–

Data are presented as mean ± standard deviation (range) or *N* (%) unless otherwise indicated

*GCSE* general certificate of secondary education, *IVR* interactive voice response

#### Score Agreement

Table 2 displays the results of the kappa and weighted kappa statistics, showing substantial to almost perfect levels of agreement across the five items based on the interpretation guidelines (i.e., kappa = 0.61–0.80 substantial; kappa > 0.81 almost perfect) from Landis and Koch [15]. One notable exception is the weighted kappa result for the self-care item, showing moderate agreement (i.e., kappa = 0.41–0.60). This result was driven by a single participant choosing a response of 1 on the first administration and a response of 5 during the second administration on the self-care item.

**Table 2:** Paper test–retest kappa and weighted kappa

Domain	Kappa	Weighted kappa
Mobility	0.661 (0.131)	0.888 (0.072)
Self-care	0.766 (0.163)	0.538 (0.356)
Usual activities	0.740 (0.100)	0.882 (0.142)
Pain/discomfort	0.819 (0.070)	0.909 (0.138)
Anxiety/depression	0.834 (0.071)	0.927 (0.098)

Figures in parentheses represent standard errors

#### Continuous Scores and Level of Agreement

Table 3 displays the means, mean differences, and ICCs for the index and EQ VAS scores from the test–retest administrations. The means for the index were approximately 0.860 with an SD of approximately 0.160. The means for the EQ VAS were approximately 82.0 with an SD of approximately 16.0. For the purposes of deriving comparison values for the three-period crossover assessments, a half SD of 0.080 for the index and 8.0 for the EQ VAS was used. Hence, the equivalence intervals for mean differences were set as − 0.040 to 0.040 for the index and − 4.0 to 4.0 for the EQ VAS. Both the index and the EQ VAS scores showed mean differences near zero and ICC values > 0.90. The thresholds for denoting equivalence on the ICC for the three-period comparisons are ICC lower 95% CI ≥ 0.911 for the index and ≥ 0.940 for the EQ VAS.

**Table 3:** Paper test–retest mean differences and intraclass correlation coefficients

Variable	Mean ± SD	Mean difference (95% CI)	ICC (95% CI)
Index: time 1	0.860 ± 0.155	0.003 (− 0.011 to 0.017)	0.946 (0.911–0.967)
Index: time 2	0.857 ± 0.168		
EQ VAS: time 1	81.550 ± 16.384	− 0.800 (− 1.927 to 0.327)	0.964 (0.940–0.978)
EQ VAS: time 2	82.350 ± 16.275		

*CI* confidence interval, *EQ VAS* EuroQoL visual analog scale, *ICC* intraclass correlation coefficient, *SD* standard deviation

### Paper-Handheld-Web Crossover Results

#### Sample Description

The paper–handheld–web crossover study included 60 participants: 33 females and 27 males (Table 1). The average age among all participants was 46.5 ± 12.6 years (range 20–79). Participants had completed either an undergraduate or postgraduate degree (*n* = 29) or attended college/technical college (*n* = 14). Most participants (*n* = 32) were English/Welsh/Scottish/Northern Irish/British, four were Black/African/Caribbean/Black British, seven were from “other” ethnic groups, and the remaining 17 participants were distributed among various ethnic groups at rates ≤ 5%.

#### Continuous Scores and Level of Agreement

The means ± SD for the index were 0.834 ± 0.165, 0.830 ± 0.166, and 0.828 ± 0.180 for the paper, handheld, and web modes of data collection, respectively. The means ± SD for the EQ VAS were 78.65 ± 16.016, 78.600 ± 16.240, and 78.533 ± 16.168 for the paper, handheld, and web modes of data collection, respectively. Table 4 displays the mean differences and ICCs for the index and EQ VAS scores from the paper–handheld–web administrations. All of the ICC point estimates for both the index and the EQ VAS were > 0.950. The mean differences for the index were wholly contained in the equivalence intervals of − 0.040 to 0.040. The mean differences for EQ VAS were also contained within the equivalence intervals of − 4.0 to 4.0. No significant mode-by-order interactions were present in either ANOVA model. The ICC 95% lower CIs for the index and EQ VAS scores showed values above the thresholds for denoting equivalence, namely ≥ 0.911 for the index and ≥ 0.940 for the EQ VAS.

**Table 4:** Paper–handheld–web mean differences and intraclass correlation coefficients

Variable	Mean difference (95% CI)	Mode × order interaction	ICC (95% CI)
Index: paper–handheld	0.004 (− 0.009 to 0.017)	*p* = 0.757	0.952 (0.921–0.971)
Index: paper–web	0.006 (− 0.006 to 0.019)		0.964 (0.941–0.978)
Index: handheld–web	0.003 (− 0.008 to 0.014)		0.970 (0.950–0.982)
EQ VAS: paper–handheld	0.050 (− 0.694 to 0.794)	*p* = 0.165	0.985 (0.975–0.991)
EQ VAS: paper–web	0.117 (− 0.248 to 0.482)		0.996 (0.993–0.997)
EQ VAS: handheld–web	0.067 (− 0.528 to 0.661)		0.991 (0.984–0.994)

*CI* confidence interval, *ICC* intraclass correlation coefficient

### Paper-Handheld-Tablet Crossover Results

#### Sample Description

The paper–handheld–tablet crossover study included 60 participants: 29 females and 31 males (Table 1). The average age among all participants was 43.9 ± 14.0 (range 22–82). Most participants (*n* = 44) had completed either an undergraduate or postgraduate degree, and seven participants had attended college/technical college. In total, 22 participants were English/Welsh/Scottish/Northern Irish/British, five were Black/African/Caribbean/Black British, six identified as mixed/multiple ethnic groups, four were Asian/Asian British, 17 selected “other,” and the remaining six participants were distributed among various ethnic groups at rates ≤ 5%.

There was one instance of missing data in the paper–handheld–tablet study. This participant had missing tablet responses and was allocated to the order of completion paper–handheld–tablet. Hence, only 59 participants are included in the analyses of the tablet data.

#### Continuous Scores and Level of Agreement

The means ± SD for the index were 0.874 ± 0.133, 0.873 ± 0.135, and 0.878 ± 0.129 for the paper, handheld, and tablet modes of data collection, respectively. The means ± SD for the EQ VAS were 81.661 ± 15.911, 80.847 ± 16.841, and 81.475 ± 16.912 for the paper, handheld, and tablet modes of data collection, respectively. Table 5 displays the mean differences and ICCs for the index and EQ VAS scores from the paper–handheld–tablet administrations. All of the ICC point estimates for both the index and the EQ VAS were ≥ 0.980. The mean differences for the index were wholly contained in the equivalence intervals of − 0.040 to 0.040. The mean differences for EQ VAS were also contained within the equivalence intervals of − 4.0 to 4.0. No significant mode-by-order interactions were present in either ANOVA model. The ICC 95% lower CIs for the index and EQ VAS scores showed values above the thresholds for denoting equivalence, namely ≥ 0.911 for the index and ≥ 0.940 for the EQ VAS.

**Table 5:** Paper–handheld–tablet mean differences and intraclass correlation coefficients

Variable	Mean difference (95% CI)	Mode × order interaction	ICC (95% CI)
Index: paper–handheld	0.001 (− 0.002 to 0.005)	*p* = 0.926	0.996 (0.993–0.997)
Index: paper–tablet	− 0.004 (− 0.009 to 0.002)		0.989 (0.981–0.993)
Index: handheld–tablet	− 0.005 (− 0.011 to 0.001)		0.983 (0.972–0.990)
EQ VAS: paper–handheld	0.807 (0.144 to 1.471)	*p* = 0.910	0.987 (0.977–0.992)
EQ VAS: paper–tablet	0.189 (− 0.592 to 0.970)		0.984 (0.973–0.990)
EQ VAS: handheld–tablet	− 0.619 (− 1.478 to 0.241)		0.980 (0.967–0.988)

*CI* confidence interval, *ICC* intraclass correlation coefficient

### Paper–Web–Interactive Voice Response Crossover Results

#### Sample Description

The paper–web–IVR crossover study included 61 participants: 48 females and 13 males (Table 1). The average age among all participants was 49.9 ± 15.8 years (range 18–77). Most participants (*n* = 32) had completed either an undergraduate or postgraduate degree, and 11 participants had attended college/technical college. In addition, most participants (*n* = 53) were English/Welsh/Scottish/Northern Irish/British, whereas the remaining eight were distributed among various ethnic groups at rates ≤ 10%.

#### Continuous Scores and Level of Agreement

The means ± SD for the index were 0.732 ± 0.270, 0.733 ± 0.273, and 0.737 ± 0.258 for the paper, web, and IVR modes of data collection, respectively. The means ± SD for the EQ VAS were approximately 72.072 ± 22.282, 72.133 ± 22.095, and 72.031 ± 22.103 for the paper, web, and IVR modes of data collection, respectively. Table 6 displays the mean differences and ICCs for the index and EQ VAS scores from the paper–web–IVR administrations. All of the ICC point estimates for both the index and the EQ VAS were ≥ 0.982. The mean differences for the index were wholly contained in the equivalence intervals of − 0.040 to 0.040. The mean differences for EQ VAS were also contained within the equivalence intervals of − 4.0 to 4.0. No significant mode-by-order interactions were present in either ANOVA model. The ICC 95% lower CIs for the index and EQ VAS scores showed values above the thresholds for denoting equivalence, namely ≥ 0.911 for the index and ≥ 0.940 for the EQ VAS (Table 6).

**Table 6:** Paper–web–interactive voice response mean differences and intraclass correlation coefficients

Variable	Mean difference (95% CI)	Mode × order interaction	ICC (95% CI)
Index: paper–IVR	− 0.005 (− 0.015 to 0.005)	*p* = 0.552	0.989 (0.981–0.993)
Index: paper–web	− 0.001 (− 0.012 to 0.009)		0.990 (0.983–0.994)
Index: web–IVR	− 0.004 (− 0.017 to 0.010)		0.982 (0.969–0.989)
EQ VAS: paper–IVR	0.067 (− 0.392 to 0.526)	*p* = 0.147	0.997 (0.994–0.998)
EQ VAS: paper–web	− 0.059 (− 0.434 to 0.316)		0.998 (0.996–0.999)
EQ VAS: web–IVR	0.126 (− 0.408 to 0.661)		0.996 (0.993–0.997)

*CI* confidence interval, *ICC* intraclass correlation coefficient, *IVR* interactive voice response

### Handheld–Tablet–Web Crossover Results

#### Sample Description

The handheld–tablet–web crossover study included 60 participants: 35 females and 25 males (Table 1). The average age among all participants was 38.2 ± 13.6 years (range 18–72). Most participants (*n* = 33) had completed either an undergraduate or postgraduate degree, and nine participants had attended college/technical college. In total, 26 participants were English/Welsh/Scottish/Northern Irish/British, six were Black/African/Caribbean/Black British, five were from mixed/multiple ethnic groups, nine participants selected “other,” and the remaining 14 participants were distributed among various ethnic groups at rates ≤ 5%.

There was one instance of missing data in the handheld–tablet–web study. This participant had missing tablet responses and was allocated to the order of completion web–handheld–tablet. Hence, only 59 participants are present in the analyses of the tablet data.

#### Continuous Scores and Level of Agreement

The means ± SD for the index were 0.860 ± 0.153, 0.860 ± 0.152, and 0.860 ± 0.147 for the handheld, tablet, and web modes of data collection, respectively. The means ± SD for the EQ VAS were 82.220 ± 12.861, 82.610 ± 12.668, and 82.288 ± 12.904 for the handheld, tablet, and web modes of data collection, respectively. Table 7 displays the mean differences and ICCs for the index and EQ VAS scores from the handheld–tablet–web administrations. All of the ICC point estimates for both the index and the EQ VAS were ≥ 0.960. The mean differences for the index were wholly contained in the equivalence intervals of − 0.040 to 0.040. The mean differences for EQ VAS were also contained within the equivalence intervals of − 4.0 to 4.0. No significant mode-by-order interactions were present in either ANOVA model. The ICC 95% lower CIs for the index and EQ VAS scores showed values above the thresholds for denoting equivalence, namely ≥ 0.911 for the index and ≥ 0.940 for the EQ VAS.

**Table 7:** Handheld–tablet–web mean differences and intraclass correlation coefficients

Variable	Mean difference (95% CI)	Mode × order interaction	ICC (95% CI)
Index: handheld–tablet	0.000 (− 0.006 to 0.006)	*p* = 0.660	0.990 (0.983–0.994)
Index: handheld–web	0.000 (− 0.010 to 0.009)		0.972 (0.953–0.983)
Index: tablet–web	0.000 (− 0.011 to 0.010)		0.966 (0.943–0.979)
EQ VAS: handheld–tablet	− 0.393 (− 0.735 to − 0.051)	*p* = 0.389	0.995 (0.990–0.997)
EQ VAS: handheld–web	− 0.031 (− 0.800 to 0.737)		0.971 (0.952–0.983)
EQ VAS: tablet–web	0.361 (− 0.305 to 1.027)		0.978 (0.963–0.987)

*CI* confidence interval, *ICC* intraclass correlation coefficient

## Discussion

This study aimed to provide evidence regarding the measurement equivalence of data collected via various data collection modes (i.e., paper, handheld, tablet, IVR, and web) for the EQ-5D-5L. The analytical strategy employed in this study conformed to the recommendations of the ISPOR ePRO task force regarding the evidence needed to support measurement equivalence [1]. As stated in the task force recommendations, electronic modes of administration should not be held to a higher standard than the original paper-based format. Hence, the paper-based test–retest component was used to set the thresholds by which the three-period crossover designs were evaluated for equivalence. For the purposes of deriving comparison values for the three-period crossover assessments, a half SD of 0.080 for the index and 8.0 for the EQ VAS were used from the test–retest data. Hence, the equivalence intervals for mean differences were set as − 0.040 to 0.040 for the index and − 4.0 to 4.0 for the EQ VAS. The thresholds for denoting equivalence on the ICC for the three-period comparisons were ICC lower 95% CI ≥ 0.911 for the index and ≥ 0.940 for the EQ VAS.

The mean difference CIs for the EQ-5D-5L index scores and EQ VAS scores reflected equivalence of the means across all modes, as the CIs were wholly contained inside the equivalence interval. Further, the ICC 95% lower CIs for the index and EQ VAS scores showed values above the thresholds for denoting equivalence across all comparisons in each sample. No significant mode-by-order interactions were present in any ANOVA model. Overall, the comparisons of the paper version of the EQ-5D-5L with the screen-based and phone-based versions provided substantial evidence supporting the measurement equivalence of these modes of administration.

However, these results have limitations, including a study sample that may lack generalizability to samples that may be enrolled in clinical trials. Because this sample was recruited from the UK general population, it is likely not representative of participants with specific conditions or diseases who enroll in clinical studies, despite at least 50% of participants having comorbid conditions. In addition, the study design, which included a limited amount of time between administrations (i.e., 30 min), may have introduced carryover, or memory, effects. The brevity and sole inclusion of the EQ-5D-5L in this study is a likely source of bias resulting in inflated agreement among the scores produced on each mode. While the impact and magnitude of carryover effects cannot be statistically estimated, it does seem reasonable to conclude that the high ICCs observed were partially a result of the participants’ ability to recall their responses from the prior administration of the EQ-5D-5L. As a practical limitation of this study, it is unlikely that the EQ-5D-5L would be administered to participants at 30-min intervals in a clinical trial, as operationalized in this study. Hence, the mean differences and ICC estimates are likely to show greater variability and lower agreement in studies comparing EQ-5D-5L scores across more distal time points. Because of these limitations, these results should be considered in the context of the study design. We do not recommend these results be used as threshold values in other studies assessing the EQ-5D-5L.

## Conclusion

The evidence presented here, when taken in totality, supports the stability of the paper-based EQ-5D-5L as well as the measurement equivalence of various electronic implementations of the EQ-5D-5L with the original paper mode and the other electronic modes.

## Data Availability

The data that support the findings of this study are available from EuroQol Research Foundation, but restrictions apply to the availability of these data, which are not publicly available. However, data are available from the authors upon reasonable request and with permission of the EuroQol Research Foundation. The authors can confirm that relevant data are included in the article.
